# Working Memory Models and Measures in Language and Bilingualism Research: Integrating Cognitive and Affective Perspectives

**DOI:** 10.3390/brainsci12060729

**Published:** 2022-06-01

**Authors:** Zhisheng (Edward) Wen, Mark Feng Teng, Lili Han, Yong Zeng

**Affiliations:** 1 Faculty of Languages and Translation, Macao Polytechnic University, Macau SAR 999078, China; hanlili@upm.edu.mo; 2 Institute of Advanced Studies in Humanities and Social Sciences, Beijing Normal University, Zhuhai 519087, China; markteng@bnu.edu.cn; 3 Concordia Institute for Information Systems Engineering, Concordia University, Montreal, QC H9R 5X7, Canada; yong.zeng@concordia.ca

**Keywords:** working memory, affective working memory, language, bilingualism, emotion regulation, *N-back* task

## Abstract

Although emotional or affective working memory (WM) is quite well established in general psychology, not much research has looked into its potential implications for the language sciences and bilingualism and second language acquisition (SLA) research until recently. To fill this gap, this paper aims to propose that WM has not just cognitive implications, but its affective dimension may also make complementary and unique contributions to language and bilingualism/SLA research. Towards this end, we first briefly synthesize the cognitive views of WM conceptions and assessment procedures in the current language sciences and bilingualism/SLA research. Next, we turn to discuss the theoretical models and assumptions of affective WM and explore their theoretical implications for bilingualism/SLA research based on emerging empirical evidence. Then, we propose a conceptual framework integrating cognitive and affective WM perspectives and further provide guidelines for designing affective WM span tasks that can be used in future affective WM–language research, focusing on the construction procedures of several emotion-based affective WM span tasks (e.g., the emotional reading span task, the emotional operation span task, and the emotional symmetry span task) as examples. Overall, we argue that affective feelings are also an integral part of the mental representations held in WM and future research in the language sciences and bilingualism/SLA should incorporate both cognitive and affective WM dimensions.

## 1. Introduction

Working memory (WM) generally refers to our ability to ‘mentally maintain information in an active and readily accessible state while concurrently and selectively processing new information’ [1]. Since its inception in the 1960s (e.g., [2]), WM has become a buzzword concept permeating a broad range of disciplines in the cognitive sciences (cf. [3,4,5]), straddling psychology, linguistics, neuroscience, human–computer interactions, anthropology, and philosophy as well as the more applied domains of human cognition and communication, such as cognitive development, education, and language learning/teaching. The implications of WM capacity and executive functions are pervasive and consequential for constraining and shaping everyday cognitive activities in language comprehension, arithmetic, reasoning, and many other cognitive tasks [6].

Affective WM (AWM), also called emotional WM, can be defined as the ability to successfully deploy WM in emotionally stressful contexts [7]; cf. [8]). However, previous theoretical and empirical studies on WM have predominantly focused on exploring its structure and functions in human cognition from multiple theoretical perspectives [9], while its emotional connotation, or affective WM, has received much less attention in general psychology and neuroscience, not to mention more practical domains such as language learning and bilingual development. In the realms of the WM–language enterprise [10,11,12,13], most previous and current research has adopted cognitive WM models (e.g., those listed in [4,14,15]) as conceptual and theoretical frameworks to investigate the putative effects of WM with a view to investigating the overall or specific implications of multiple WM components (e.g., Baddeley’s model) and executive control functions (e.g., Cowan’s and Engle’s model) for miscellaneous domains of language acquisition and bilingual processing [16]. In sharp contrast, research probing the role of affective WM (AWM) in language and/or bilingualism/SLA has been meager until recently [17]; see also [18].

To fill the gap, this paper proposes not only that WM in language and bilingualism/SLA has cognitive implications but also that its affective dimensions should not be neglected. It is argued that affective WM may hold great promise in making distinctive and unique theoretical and methodological contributions to future SLA/bilingualism research. Towards this goal, we first summarize the cognitive approaches to WM conceptions and the assessment procedures implemented in current language and bilingualism/SLA research, unearthing some remaining issues besetting theory and methodology. Following these, we turn to elaborate on the construct of affective WM and integrate it with cognitive WM perspectives on bilingualism/SLA studies. To facilitate future studies, we also propose some practical guidelines for constructing a series of affective WM span tasks that can be readily implemented in future affective WM–language research as complementary to current cognitive paradigms. Overall, we argue that both cognitive and affective WM dimensions provide important, albeit distinctive, insight into the WM–language nexus [19,20], and therefore, future research in the language sciences and bilingualism/SLA should incorporate both perspectives.

## 2. Cognitive WM Models and Measures in Language and Bilingualism/SLA

WM, as the primary memory (cf. [21]), plays a fundamental role in multiple facets of human cognitive life, including language learning and processing [10,22,23]. Research on WM drawing on the integration of a large number of empirical investigations of typical and nontypical participants constituted a major source of and the catalyst for the formulation of the seminal multicomponent model by Baddeley and colleagues [10,24]. This multicomponent model of WM by Baddeley [25,26] has thus become the most widely cited framework across multiple disciplines, including language and bilingualism/SLA research [27,28,29].

Among the four components as conceived in Baddeley’s fractionated WM model, the phonological loop (or phonological WM; [20]), comprising a phonological store and an articulatory rehearsal mechanism, has been postulated to play an instrumental role in the storage and processing of novel phonological forms [30], rendering it the ‘*language learning device*’ [25]( see also [31]). Inspired by this hypothesis, numerous empirical studies adopting diverse research methodologies have corroborated the positive links between phonological WM (measured by some simple storage-focused versions of memory-recall tasks such as the digit span and the nonword recognition/repetition span; [32]) and a broad range of language-learning domains among both typical and non-typical developmental participants [33]. These language domains include, most obviously, the acquisition and development of lexical knowledge or word learning in both L1 and L2 (e.g., [30,34,35,36]). Longer linguistic units, such as phrases or multi-unit formulaic chunks, have been found to rely on phonological WM as well (e.g., [37,38,39,40]), though its role in morpho-syntax or grammatical structure is debatable (largely depending on the different epistemological stances on the very definition of ‘grammar’ and the specific models of language; cf. [41,42]).

Additionally, phonological WM, with its putative articulatory rehearsal mechanism, has also been found to be closely related to oral fluency and development at the early stages of language learning in both L1 and L2 (e.g., [43,44,45]). As such, the pivotal role of phonological WM as conceived in Baddeley’s multicomponent model in storage- and sound-based language acquisitional and developmental aspects is now firmly established (see also [46,47,48]. In contrast to phonological WM, other WM components, such as visuospatial WM and the episodic buffer in Baddeley’s model, have received much less enthusiasm among second-language researchers [22,49], nor have the linguistics- and psycholinguistics-oriented components, such as semantic WM and orthographic WM, been vigorously researched (e.g., [50]). In more recent years, though, Baddeley and colleagues [26] have explored the role of the episodic buffer in binding visual information such as objects. This emerging trend will hopefully renew momentum towards investigating the implications of visuospatial WM and the episodic buffer for sentence recall (e.g., [51,52]) and for following spoken instructions (i.e., the enactment effect) [53,54].

Despite the paucity of empirical studies directly probing the central executive in language and SLA/bilingualism and adopting Baddeley’s structural view of WM [22], other functional WM models have witnessed increasing theoretical and empirical investigations into the individual variations in the executive control or attentional control aspects of WM [55]. Two theoretical frameworks are gaining increasing prominence in this line of inquiry, namely the embedded-processes model by Cowan [9] and the executive control or attentional control paradigm touted by Engle [56,57]. Though controversies and debates still linger over the sources of such inherent variations [1] and constituent sub-processes, executive WM conceived this way (i.e., EWM in [20]) is generally operationalized and measured by more cognitively demanding dual-processing (e.g., storage plus manipulation) span tasks in both the psychological and language sciences [58,59], including bilingualism/SLA research [60,61].

These ‘complex’ versions of executive WM span tasks include the seminal reading span task that measures sentence judgment accuracy and serial recall of final words [62], the scoring procedures of which have been further refined by Waters and Caplan [63] to also take into account participants’ reaction time for the judgment component. Other formats of the complex memory span tasks are gaining popularity [60], including the domain-general operation span task, which taxes participants’ dual-processing ability to solve arithmetic equations and recall final items [64] as a way to avoid confounding linguistic proficiency in the reading span paradigm. Another format, i.e., the *N-back* task, is commonly applied in both neuropsychology (e.g., cognitive and WM training); [65]) and language-cum-SLA/bilingualism research ([66]), though its underlying mechanism is far more controversial [67].

Regarding the effects of these executive aspects of WM (i.e., EWM [20]), empirical studies have pointed to their close links with cognitively demanding language processes and activities both online and offline during L1 reading and parsing (e.g., ambiguity resolutions, morphological and grammatical processing, e.g., [68,69]) and L2 sub-skills such as listening, speaking, reading, writing, and bilingual interpreting (e.g., [70,71,72]).

The most recent trend of executive functions related to WM is the ‘*unity and diversity*’ framework [73,74] that is making its inroads into language and SLA/bilingualism research. Scholars endeavor to explore the componential and separate effects of these executive functions (e.g., updating, task switching, and inhibitory control) on L1 and L2 learning and development [16]. Among the three key executive functions, memory updating and inhibitory control, as measured respectively by the running memory span task [75] and the *N-back* task [76], have garnered increasing attention in SLA (e.g., [77]) and task-based language-teaching research [18,28,78]. Similarly, the well-articulated attentional control paradigm by Engle and colleagues [79,80] has been cited widely in the language sciences (cf. [81]) and interpreting models [82].

Overall, an increasing body of empirical studies as discussed in comprehensive narrative reviews [15,20] and meta-analytic surveys (e.g., [69,71,83]; see also [47,84]) has reinforced the positive links between the putative WM components (esp., phonological WM and executive WM) and executive control functions (e.g., updating, task-switching, inhibitory control, attentional control, etc.) as they relate to nuanced language-learning domains and skills in L1 and L2. These emerging patterns, when effectively integrated and synthesized further, lend theoretical support and empirical evidence to the formulation of an integrated cognitive account that portrays the intricate relationships between WM components and functions on the one hand, and language as well as bilingualism/SLA on the other. These hypothetical links thus culminate in the phonological/executive (P/E) model [12,20,85,86].

Moreover, as the theoretical models of WM evolve, WM measurement procedures are also evolving [61]. As such, the integrated account of WM and language/SLA has also identified and regrouped the array of WM span tasks currently available from cognitive psychology and neuroscience (e.g., [58,59]). Specifically, the P/E model [20] has stipulated that the simple (storage-only) versions of memory span tasks (e.g., digit span, nonword repetition span) are approximating phonological WM, while the complex (storage-plus-processing) versions of memory span tasks serve as a proxy for executive WM. Furthermore, in alignment with the emerging ‘*unity and diversity*’ framework, finer-grained sub-process-oriented WM measures (e.g., storage, articulatory rehearsal, updating, task switching, inhibitory control) are in place to tap into granulated executive or attentional control mechanisms and functions that impact language and bilingualism/SLA domains.

To sum up, previous and current empirical studies adopting cognitive WM perspectives have pointed to the positive, albeit distinct, roles of phonological WM and executive WM as they relate to specific SLA domains and L2 sub-skill learning ([20]; cf. [87] for semantic WM vs. phonological and orthographic WM). For example, Linck et al. [71] reported an overall (population) effect size of 0.255 between WM and L2 processing and products. Such an effect size is small, but the reasons behind this finding are still unclear. On the one hand, it is possible that WM may be a necessary but not an essential factor in SLA. On the other hand, such a small effect size can also be partly due to the differences in methodology such as the inconsistency of WM span tasks across the empirical studies [60]. Though an enormous number of studies have adopted these dominant cognitive paradigms to investigate WM effects and their potential consequences for language and bilingualism/SLA, they are not the only approaches and are not readily embraced by all linguists and psycholinguists. It is even true that in ‘mainstream’ theoretical linguistics and psycholinguistics, for example (cf. [88]), the role of WM or general memory as a whole is generally downplayed and marginalized, sometimes to the extent of negligibility (e.g., [89,90]; cf. [91]).

For example, Chomsky [92] has unequivocally speculated that the language acquisition device (LAD; or universal grammar) should be unaffected by such ‘grammatically irrelevant conditions as memory limitations, distractions, shifts of attention and interest, and errors (random or characteristic) in applying knowledge of the language in actual performance’. (p. 3) Contrary to Chomsky’s dominant view in mainstream linguistics, other processing- and performance-oriented theoretical linguists (e.g., [41,93]) as well as emergentist-oriented (e.g., [94]) and typological or dependency grammar–oriented linguists ([95]; cf. [96]), have all attached great emphasis to the role of WM, holding the view that WM limitations are part and parcel of the language parser (or the language device; [84,91]). It is even claimed that WM limitations play a pervasive, albeit sometimes ‘hidden’, role in key domains of language design, acquisition, and processing of linguistic structures and constructions ranging from phonology to grammar and discourse ([94]; cf. [97]). Other psycholinguists-cum-neuroscientists (e.g., [29]) have recently advocated studies of domain-specific WM components such as semantic WM and orthographic WM alongside the prevailing phonological WM, derived from distinct neural correlates from neuropsychological evidence (e.g., [29,87]). On a cautionary note, some psycholinguists (e.g., [90]) have not ruled out the possibility that WM may be no more than an ‘emergent’ (parasitic) by-product of language comprehension and production (cf. [89,98].

## 3. Integrating Cognitive WM with Affective WM in Language and Bilingualism/SLA

### 3.1. The Theoretical Underpinnings

Despite its predominant reliance on cognitive WM models and span tasks in previous language and bilingualism/SLA research, the emotional or affective component of WM (AWM) has also received attention in general psychology, which has gradually made its forays into bilingualism research in recent years (e.g., [17]). In line with this new trend, we may also need to acknowledge and consider the potential interactions between cognition and emotion implicated in these traditional cognitive WM models and WM span tasks [58,59].

Recent years have witnessed a growing interest in exploring the effects of emotions such as mood and affect on WM [99] as well as their potential impacts on bilingual processing [17]. Similarly, emotional or affective constructs have been intensely investigated in current applied linguistics and SLA, including both well-established individual differences (IDs) concepts such as motivation, anxiety, and willingness to communicate as well as emerging constructs such as enjoyment, flow, grit, and even boredom [100]. It is not difficult to imagine that, when L2 learners are highly anxious about an upcoming reading span task of WM, a natural consequence may be that the participants may find themselves unable to maintain mental focus on the sentential judgment, not to mention the recall part, and thus their performance on either accuracy or reaction time will be affected (most likely negatively in these cases). Under this circumstance, it may be the emotional and affective state (i.e., anxiety) rather than their cognitive WM capacity that impairs performance in maintaining and processing information necessary for the completion of the WM span task.

Interpreted this way, the participants’ emotions (e.g., mood, anxiety, happiness, fear, etc.) may serve to modulate their WM capacity [99]. For example, consider a teenage student who is retrieving and processing information for a computer project but suddenly breaks his newly purchased iPhone. The student might focus on the frustration that arose from the broken iPhone, and thus, the attention to the original task (the computer project) became distracted as the student was likely to maintain this broken-hearted feeling in mind until the intensity diminished. On another occasion, if the same student was just promised to receive a long-desired gift (e.g., the latest MacBook) if he can deliver an excellent project result, such a motivation would likely increase the student’s WM capacity. Scenarios like these may be referred to as affective WM, a specific type of WM that maintains and works with the emotional or feeling states such as pleasure, pain, fear, anger, and other common emotions (e.g., [101,102,103]).

That is to say, although it is generally accepted that WM is an important instrument for cognition, the role of emotion and affect in WM is also tenable, albeit debatable. One could further argue that emotional stimuli have been seen as an attentional workload, which explains the impact of emotional valence (e.g., [50,104,105]). However, research has shown that positive affect (e.g., happiness) improves WM, particularly the controlled processing in WM (e.g., [106,107,108]). Naturally, this leads to the question: should we consider that affective features influence WM abilities or that WM has an affective component? While further evidence is necessary for the research community to disclose the underlying mechanism behind the impact of emotions on WM capacity, we can fairly safely hypothesize that positive emotions (e.g., happiness, excitement, joy, etc.) may enhance our WM, while negative emotions (e.g., anxiety, fear, or sadness, etc.) may reduce our WM capacity (e.g., [109]). However, we still need to determine under which conditions these positive or negative effects may emerge and to what extent they interact with cognitive WM.

**Figure 1:**
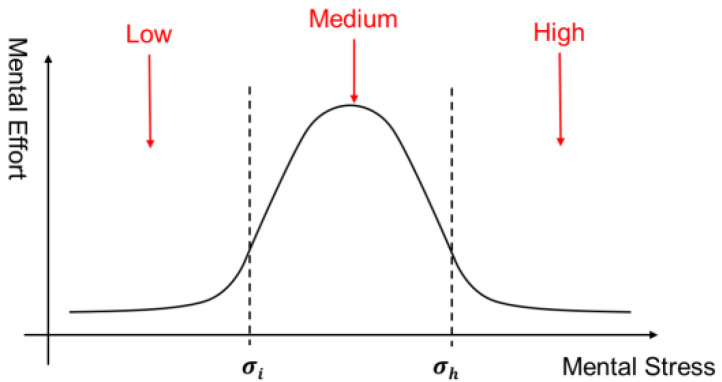
Mental Stress-Effort Model [111].

To provide an answer to this question, we draw on a theoretical model that simulates the dynamic interactions between human cognition and emotion during task implementation, i.e., the TASKS framework [110], to propose a conceptual framework that integrates cognitive and affective WM perspectives. The TASKS framework (encompassing task, affect, skills and knowledge, and stress) postulates that an individual’s performance (behavior) on any cognitive task depends on the individual’s cognitive abilities (in this case, WM capacity) and the mental effort put into the task. Furthermore, the mental effort that an individual can put into a task is related to the mental stress triggered by the task through an inverted U-shaped curve, as shown schematically in Figure 1. Based on Figure 1, it can be hypothesized that low- and high-level mental stresses would produce low-level mental efforts. In contrast, medium-level mental stress results in optimal-level mental efforts. For an easy cognitive task, a low mental effort could be sufficient to produce high performance with low mental stress, whereas a low mental effort for a complex task implies poor performance.

Nguyen and Zeng [112,113] qualitatively defined human mental stress as the ratio of perceived workload over mental capacity, as described in the equation below.

$$Mental\;Stress=\frac{Perceived\;Workload}{Mental\;Capacity}\;=\frac{Perceived\;Workload}{\left(Knowledge+Skills\right)*Affect}$$

In the equation, knowledge, skills, and affect are three key factors determining the human mental capacity to tackle a perceived workload related to a given task. The workload is an external load exerted on an individual, which can be associated with the cognitive complexity of the task. The amount of external workload constitutes the most direct source of mental stress. Both knowledge (e.g., long-term knowledge base) and skills (e.g., logic thinking, executive control processes) form cognitive capability, whereas affect refers to affective capability (e.g., emotion regulation/coping) and determines how much of an individual’s cognitive capability can be activated and harnessed to complete a given task.

By the same token, when cognitive WM functions are maintaining and manipulating verbal (or visual) representations, they can also operate on emotional representations, thus constituting affective WM (AWM). Distinct from cognitive WM, emotional or affective WM (AWM) has several unique features [102]. First of all, it maintains a mental representation, i.e., the subjective emotional feeling. In particular, the emotional connotation of task-relevant information is likely to influence an individual’s behaviors or actions, even in the absence of immediate emotion-eliciting stimuli (e.g., fearful feelings). Second, the maintained representation in affective WM is modulating learners’ subsequent goals either beneficially, neutrally, or detrimentally (e.g., happiness vs. sadness; [107]). Third, affective WM involves active maintenance of a feeling or emotion, rather than a passive experience. Finally, affective WM involves the maintenance of distributed neural representations of feelings, for which attentional control is required for active maintenance. Above all, affective WM processes exert deliberative control over the emotional feeling and affective aspects of task-relevant (and possibly even task-irrelevant) information.

In their integrative framework of affective WM, Mikels and Reuter-Lorenz [8] depict three modes of emotion–WM interactions. Each mode has general principles for the functioning of WM concerning emotional feelings. Mode 1 focuses on the positive versus negative emotions that people experience and how such emotions may influence their engagement in ongoing cognitive tasks. Mode 2 concerns the influences of cognitive WM on emotional experiences, which can either enhance or diminish emotions. Mode 3 stipulates that emotional feelings can be mental representations maintained in WM. It is thus argued that variances in mental representations constitute one part of WM and that WM functions in responding to the changes in emotional states. Put another way, Mode 1 in the framework are affect-laden stimuli that are processed and maintained by cognitive WM, while Mode 3 predominantly involves the maintenance of subjective emotional feeling states.

### 3.2. Empirical Evidence

Following these theoretical insights discussed above, we now argue that such an emotional component of WM, or affective WM, is poised to make distinctive and complementary contributions to conceptualizing and measuring WM in future empirical research in bilingualism and SLA (e.g., [18]). So far, only sporadic studies in the bilingualism field have investigated the possible transfer or facilitative effects of bilingual experience on affective WM. These studies have been conducted primarily by team members and collaborators of Bialystok’s lab [7,17,114]. For example, Janus and Bialystok et al. [17] explored the influence of bilingualism on emotional regulation (ER). They also compared such effects between monolingual healthy young children vs. bilingual counterparts. Data were collected from school-age (8 to 11 year-olds) bilingual children learning English as a second language who were of diverse L1 backgrounds, e.g., Portuguese, Filipino, or Spanish. Another group of learners was monolingual children, who served as the control group. The Emotional *Face N-back* task they adopted focused on three emotional contexts of anger, happiness, and neutrality, respectively. The results indicated that bilingual children demonstrated higher levels of accuracy than monolingual children, suggesting bilingualism advantages [115]. However, no difference was detected in the response time (RT) in the two groups under the 1-back condition, while under the 2-back condition, bilingual children demonstrated higher RTs than monolingual children, suggesting no differences in emotional regulation between language groups. Similar results were also detected among adult subjects in a follow-up study conducted by Barker and Bialystok [114].

However, as Ma et al. [7] noted, there were some contradictions in these two previous studies by Bialystok et al. between accuracy rate and RTs. Both of these studies had adopted the *Face N-back* task in emotional contexts, but it was not clear whether the influence of bilingual experience on emotional WM task was due to task-specificity or other (task-irrelevant) factors such as geography, economic status, and educational background, all of which may play a role in the positive effects [71,115]. In addition, the bilingual experience should not be treated as categorical [116] but instead should be considered as a continuous variable (similar to that of L2 proficiency) for which different levels of experience can be categorized for data analysis for understanding the fixed and random effects (see also [71,117]).

Following these arguments, Ma et al. [7] further explored the influence of bilingual experience on individuals’ emotional WM. This latest study distinguished itself from previous studies by adopting a delayed matching-to-sample task paradigm in Experiment 1 and the *N-back* task in Experiment 2. The delayed matching-to-sample task paradigm allowed the researchers to peer into participants’ ability in maintaining WM information [118,119], while the *N-back* task paradigm was a more complicated task for measuring memory updating ability besides maintaining WM information [114]. Data were collected from Chinese–English bilinguals. The results showed that proficient bilinguals have better performance on both of the two WM tasks than non-proficient bilinguals, suggesting a bilingualism advantage. It was further found that negative emotions impact the complex *N-back* tasks. Consistent with previous studies, this investigation corroborated that abundant bilingual experience is an important factor to be considered. Such experience can possibly promote the individual development of cognitive ability and enable individuals to possess cognitive advantages on WM in emotionally stressful contexts (i.e., enhanced affective WM). In addition, Ma et al. [7] also found that the background information with negative emotions could improve individuals’ performance in complex *N-back* tasks. To be more specific, under the 2-back condition, the facilitative effect of negative emotions was significantly greater than that of positive emotions and neutral emotions, and this result was consistent with accumulating evidence based on previous studies (e.g., see [120,121]). A typical example of the aforementioned results was seen in a study by Grimm et al. [122], who took advantage of the emotional *N-back* task to explore the differences in the functions of (memory) updating under different emotional expressions. The findings revealed that negative emotion words significantly facilitated the participants’ reaction in WM under the 2-back condition.

## 4. Construction of Affective WM Measures

Based on emerging patterns from the empirical studies discussed above, we can tentatively argue that the influence of emotional conditions on WM might indeed be affected by the cognitive complexity of the task (e.g., *N-back*). For example, more complex WM tasks (the 2-back) may be influenced to a larger extent (as opposed to the 1-back condition). Because more complex WM tasks entail higher cognitive load, the association between emotion and WM could be modulated based on the adjusted cognitive load. In line with these affective perspectives, we now turn to incorporate an alternative affective component of WM in constructing a new breed of WM span tasks as complementary to their cognitive counterparts. In the next section, we discuss methodological considerations and propose practical guidelines for implementing these emotion-oriented AWM span tasks in future WM–language/SLA explorations.

To begin with the most straightforward solution, AWM can be measured by the standard WM task augmented with an inserted emotional background (e.g., [114,123]). Compared with the standard or cognitive memory span tasks with no emotional background [58], emotional WM span tasks entail higher demands on cognitive operation due to the extra requirements for emotional processing [17,106,123]. Affective WM span tasks do not tap into only cognitive demands (such as storage and processing) but also can serve to examine whether and to what extent the participants can resist the interference of emotions and how effectively they can operate information processing in an emotional and social environment [17]. That is to say, the purpose of using affective WM tasks is to assess individuals’ emotion regulation (ER) abilities, problem-solving abilities, and social adaptation competence that participants may experience in real-life situations [17,123,124,125].

Applying these insights in the context of bilingualism/SLA, the perceived workload is associated with the WM task difficulties while cognitive and affective capabilities are supported by the CWM and AWM, respectively. Drawing from the above theoretical discussion (Section 3.1), it becomes plausible for bilingualism/SLA researchers to manipulate the cognitive load [64] as a means to control the degree of the difficulty levels for WM span tasks. For example, by drawing from the research design of previous studies and experimental paradigms [17,114,], three kinds of conditions of face emotional contexts—neutral, negative (sadness) and positive (happiness) emotions—can be incorporated into the traditional WM span tasks such as the *Face N-back* task [99]. Such a design has an advantage in exploring the differences in WM between bilingual people with different levels of proficiency. That is to say, during the research design of WM–language studies, we may also need to reconsider whether a rich bilingual experience is conducive to promoting only the cognitive WM in individuals, or whether such an effect could be modulated by their ability to regulate emotions, as indicated by performance in affective WM span tasks. For example, researchers may need to consider whether individuals with more bilingual experience can effectively resist the interference of executive function stimulation and maintain more advantages in WM performance. Questions like this merit empirical studies in future research.

Alternatively, as discussed in the previous section, an emotional or affective component could be added to existing cognitive WM span tasks already in use in current WM– SLA/bilingualism research [17]. We demonstrate in the following sections several new affective WM span tasks that we developed (see also [18] for preliminary discussions). We hope that the introduction of the following affective WM tasks can provide new methods to assess AWM capability in future bilingualism/SLA research.

### 4.1. The Emotional Reading Span Task (ERST)

This section elaborates on the construction process of a WM span task which is called the emotional reading span task (ERST). This task was modeled on the previous reading span task [62], which has been widely cited in both psychology and language sciences. It has been argued as a reliable measure in measuring the cognitive WM capacity to store and manipulate information in parallel [68]. The standard procedure of the RST was first to read a series of unconnected sentences aloud. The learners had to remember the final word of each sentence of the set at the same time. The end-of-sentence words in the originally presented order at the end of a set represent the storage function. The number of sentences in a set is incrementally increased. The total number of final words correctly recalled represents the participant’s span. Different from “simple span” tasks, such as the digit span and letter span, the reading span task measures both storage and executive functions of WM.

The ERST distinguished itself from the EST in that the sentences for the learners to read reflect eight types of basic emotions in human behaviors [126]. Some sample sentences are listed in the Appendix A. In total, there are 80 sentences, ranging in length from around 10 to 17 words each. Each type of emotion includes 10 sentence stimuli or prompts. The number of sentences in each set is increased. The sentence-final words to be remembered by the participants are not repeated. The participants are required to remember all end-of-sentence words in order. The item that the learners can recall correctly in the correct order is awarded one point. Any incorrect item is awarded zero points. Instructions on the task are shown on the computer screen. The task starts with practice trials to help learners familiarize themselves with the task requirements. To avoid the influence of (L2) language proficiency effects on WM, we developed two versions, a Chinese version (for Chinese native speakers) and an English version (for English native speakers). The task can be administered through the psychological program software of E-prime [127]. Currently, we are also trying to put the ERST online so that researchers can have easier access for further validation and research purposes.

### 4.2. The Emotional Operation Span Task (EOST)

In this new E-prime-based EOST, we added an emotional or affective component to the traditional operation span task [128]. Such addition of emotional components represents the theoretical conceptualization of WM as “controlled attention” in the face of distraction [129]. This task includes a total of 66 mathematical operations and 66 to-be-remembered Chinese words. The different mathematical operations are organized into 15 sets of trials. Each trial set includes a different number of operation-word strings, ranging from two to six. The order of set length is randomized. Three series of two operation-word strings are used as practice trials.

The procedure for EOST is as follows: First, the participants are presented with an operation and then an emotionally manipulated word (e.g., positive, negative, or neutral) strings, e.g., “(6/2) + 2 = 5 (Y/N?) blood”(One reviewer suggested we place the emotional word for recall at the beginning of the sentence instead of before the equation; we agree that this is an interesting suggestion, but for now, perhaps it is too early to do that and it might be better and safer to do the same as the cognitive paradigms first. That said, future research can explore the differential impacts between their initial positions vs. final positions.). They are required to read the operations aloud and decide whether the operations are correct or not by pressing the “Y” (yes) or “N” (no) button and at the same time say “Dui” (“对”; correct) or “Cuo” (“错”; wrong) aloud. A two-character Chinese emotional word then appears immediately on the screen. The participant reads aloud the Chinese word and then clicks the mouse for another operation-word string. After completing the required operation-word strings in each set, the participant is asked to recall the Chinese words in the original order. An MP3 recorder is used to record the recall.

The scoring procedure is based on the traditional operation span task [128]. A missing item or an incorrect item is scored as zero points. One point is given for a word correctly recalled in the correct order. A subject’s span is the total of the correctly recalled words. The possible span scores range from 0 to 66. The accuracy rate on the mathematical operations, based on Conway and Engle [128], is set at 85%. That said, the participant’s data is included when the accuracy rate for the mathematical equations is below 85%.

The following measures are taken to ensure the reliability and validity of this novel emotional operation span task: (a) the 66 mathematical operations are controlled at a similar difficulty level. One reason is that unequal arithmetic/mathematical ability may influence their WM [128]. In addition, we include simple mathematical equations to avoid any possible math anxiety that may occur [130]; (b) the EOST is administered on an individual basis within 15 min, thus preventing subjects from manipulating idiosyncratic strategies that may jeopardize the validity of the tests [131]; (c) the recall items, i.e., the words to be remembered after math equations, should be emotionally manipulated as positive, negative, or neutral [99], and none of these recall items are semantically associated with each other; and (d) the instructions for the EOST are made clear [132].

### 4.3. The Emotional Symmetry Span Task (ESST)

The development of E-prime-based ESST is based on the traditional symmetry span task [31]. The rationale for the symmetry span task is that when an individual has to process a set of locations encompassed within a structure, the reflection or recall of the location in more than one way reflects one’s spatial WM. Symmetry perception can be considered to reflect human cognition. Perception of symmetrical and asymmetrical patterns has been extensively investigated, suggesting that the detection of symmetry may facilitate early visual processes, e.g., figure-ground segmentation, and later processes, e.g., recognition of objects from novel viewpoints [133]. The purpose of developing ESST is to add the affective component into the assessment of visual–spatial WM.

The feature of this task is to recall sequences of emotional pictures within a matrix while performing a symmetry-judgment task. The learners are first required to do a symmetry-judgment task, serving as a distractor task. The distractor task is a set of 8 × 8 matrices with some emotional pictures. It is presented for 650 ms. Participants then decide whether the design is symmetrical about its vertical axis. About half of the patterns are symmetrical. The learners are then directed to the storage-alone session, for which they are exposed to sequences of emotional pictures appearing in a 4 × 4 grid. They need to click the correct locations in the matrix based on the preceding displaying order. Set sizes range from two to five symmetry–memory matrices per trial (for 15 trials total). The possible span scores ranged from 0 to 60. The scoring procedure is the same as for the EOST and ERST. Three series of two symmetry–emotional picture strings are used as practice trials.

## 5. Towards an Integrated Agenda for Cognitive and Affective WM in Language and Bilingualism/SLA Research

Accumulating evidence from psychology and neuroscience has converged on the conception of WM as a complex and dynamic memory system consisting of multiple components and mechanisms [4,5]. An individual depends on the comprehension of and interaction with the input from the environment to retain information about the immediate past and to act on certain goals to complete some cognitive activities. So far, a whole array of WM span tasks has been constructed in cognitive psychology and administered widely to assess possible impacts of different facets of WM functions or mechanisms on human cognitive activities such as first and second-language learning and processing [58,59]. Previously, cognitive WM tasks have been designed predominantly based on either verbal or spatial information by manipulating the stimuli in conjunction with cognitive processes tapped (storage-only simple span vs. storage-process dual processes), modality-oriented (verbal vs. visual or spatial), and language presentation modes (L1 vs. L2), thus creating some inherent limitations and caveats in interpreting or comparing results of these WM span tasks. In this paper, we propose the incorporation of an emotional or affective component into traditional cognitive WM span tasks (especially the three complex versions of the emotional reading span, operation span, and symmetry span) as providing additional and complementary insights into their potential impacts and effects of AWM. Emotion itself is likely to have a fleeting effect on WM performance and cognition. Different types of emotions (e.g., happiness vs. sadness) may have different impacts on WM capacity (e.g., [107]), which may, in turn, lead to different performance levels of language learning and processing. For example, negative emotion appears to have an impairing effect on WM. In contrast, positive emotion and motivation may enhance WM performance. All these tentative assumptions, however, still need to be confirmed with more empirical studies conducted in the bilingualism/SLA field.

Affective WM has recently been suggested as being related to learning a second or foreign language [17]. It is an emotional system responsible for integrating the different components of second or foreign language learning. Navigation between two languages requires us to hold some linguistic information in mind while, at the same time, manipulating another language. Such a process mirrors the manipulate/maintain definition of WM. Still, we face many unanswered questions. One question is how we should conceptualize and measure affective WM, and we have provided tentative solutions to this question (in Section 3 and Section 4). In the future, we need to study the role of affective WM in increasing our success in second-language acquisition, including its different effects on monolingual versus bilingual groups. Other questions include whether stronger affective WM ability improves the chances of becoming bilingual or whether learning a second or foreign language improves one’s affective aspects of WM performance. Finally, we need to learn whether the emotional effect may be chronic and whether we can assign a medium degree of malleability due to the effort needed to become bilinguals. To conclude, the potential influence of bilingual education is more profound and far-reaching, and the benefits of bilingual experience on capacities of both cognitive and emotional WM should be investigated and discussed in future language science and bilingualism/SLA research.

## Data Availability

Not applicable.

## Appendix A

**Table A1:** Example prompts for each type of emotion (Emotional Reading Span Task).

Types of Emotions	Example Prompts for Each Type of Emotion
Joy	I feel pleased because my mom praised me just now. 我妈刚刚表扬了我，我很高兴。 It is a good day with warm sunshine. 今天天气真正好，暖阳和煦。
Sadness	I feel upset because my mom criticized me just now. 我妈刚刚批评了我，我很难过。 It was a rainy day when he left me. 他离开我的那天，天空中飘洒着雨。
Surprise	I feel so surprised because my mom praised me just now, which she had never done so before. 我妈刚刚表扬了我，我很惊喜，因为她以前从未表扬过我。 It turned out to be a good sunny day instead of a rainy day by the forecast. 那天天气真好，暖阳和煦，并不是天气预报说的下雨天。
Trust	We tell each other everything. 我们之间无话不说。 Out of trust, he let her manage all the money. 出于信任，他让她管理所有的钱。
Anger	I feel angry because my mom criticized me for no reason just now. 我妈无缘无故地批评了我一顿，我很生气。 He looks so angry that he may kill her. 他看起来很生气，仿佛要把她给杀掉。
Disgust	A worm is crawling in my dish. 餐中有条虫在爬。 My friend put a spider in my quilt. 朋友把一只蜘蛛放我被子里。
Anticipation	I feel pleased because my mom may praise me later. 我感到高兴，因为我妈等会儿可能会表扬我。 Tomorrow is expected to be a good day with warm sunshine. 预计明天会是阳光和煦的好日子。
Fear	I broke my mom’s vase, and I was scared. 我打碎了我妈的花瓶，很害怕。 Living in this haunted house was full of fear. 住在这个闹鬼的房子充满了恐惧。

## References

[1] Conway A.R.A., Jarrold C., Kane M.J., Miyake A., Towse J.N. (2007). Variation in Working Memory.

[2] Miller G., Galanter E., Pribram K.H. (1960). Plans and the Structure of Behavior.

[3] Miller G. (2003). The cognitive revolution: A historical perspective. Trends Cogn. Sci..

[4] Miyake A., Shah P. (1999). Models of Working Memory: Mechanisms of Active Maintenance and Executive Control.

[5] Oberauer K., Lewandowsky S., Awh A., Brown G.D.A., Cowan N., Donkin C., Farrell S., Hitch G.J., Hurlstone M., Ma W. (2018). Benchmarks for models of working memory. Psychol. Bull..

[6] Cowan N. (2005). Working Memory Capacity.

[7] Ma X., Ma X., Li P., Liu Y. (2020). Differences in working memory with emotional distraction between proficient and non-proficient bilinguals. Front. Psychol..

[8] Mikels J.A., Reuter-Lorenz P.A. (2019). Affective working memory: An integrative psychological construct. Perspect. Psychol. Sci..

[9] Cowan N. (2022). Working memory development: A 50-year assessment of research and underlying theories. Cognition.

[10] Baddeley A.D. (2003). Working memory and language: An overview. J. Commun. Disord..

[11] Gathercole S., Baddeley A. (1993). Working Memory and Language.

[12] Wen Z., Schwieter J., Schwieter J., Wen Z. (2022). Towards an integrated account of working memory and language. The Cambridge Handbook of Working Memory and Language.

[13] Wen Z., Baddeley A., Cowan N. (2022). Working Memory in First and Second Language.

[14] Baddeley A.D., Hitch G.J., Allen R., Logie R.H., Camos V., Cowan N. (2021). A multicomponent model of working memory. Working Memory: State of the Science.

[15] Schwieter J., Wen Z. (2022). The Cambridge Handbook of Working Memory and Language.

[16] Bunting M.F., Wen Z., Logie R., Wen Z., Gathercole S., Cowan N., Engle R. (2022). Working memory in language and bilingual development. Memory in Science for Society.

[17] Janus M., Bialystok E. (2018). Working memory with emotional distraction in monolingual and bilingual children. Front. Psychol..

[18] Wen Z., Teng F., Han L., Lambert C., Aubrey S., Bui G. (2022). Affective working memory in task-based language teaching research. The Affective Dimension of Task-Based Language Teaching Research.

[19] Wen Z. (2012). Working memory and second language learning. Int. J. Appl. Linguist..

[20] Wen Z. (2016). Working Memory and Second Language Learning: Towards an Integrated Approach.

[21] James W. (1890). Principles of Psychology.

[22] Baddeley A.D., Wen Z., Mota M., McNeill A. (2015). Working memory in second language learning. Working Memory in Second Language Acquisition and Processing.

[23] Baddeley A.D. (2017). Modularity, working memory and language acquisition. Second Lang. Res..

[24] Baddeley A.D. (2021). Developing the concept of working memory: The role of neuropsychology. Arch. Clin. Neuropsychol..

[25] Baddeley A.D., Gathercole S., Papagno C. (1998). The Phonological Loop as a Language Learning Device. Psychol. Rev..

[26] Baddeley A.D., Hitch G.J., Allen R.J. (2009). Working memory and binding in sentence recall. J. Mem. Lang..

[27] Ellis R. (2005). Planning and Task Performance in a Second Language.

[28] Ellis R., Skehan P., Li S., Shintani N., Lambert C. (2020). Task-Based Language Teaching: Theory and Practice.

[29] Martin R.C. (2021). The critical role of semantic working memory in language comprehension and production. Curr. Dir. Psychol. Sci..

[30] Service E., De Borba E., Lopez-Cormier A., Horzum M., Pape D. (2022). Short-Term Memory for Auditory Temporal Patterns and Meaningless Sentences Predicts Learning of Foreign Word Forms. Brain Sci..

[31] Unsworth N., Redick T.S., Heitz R.P., Broadway J.M., Engle R.W. (2009). Complex working memory span tasks and higher-order cognition: A latent-variable analysis of the relationship between processing and storage. Memory.

[32] Gathercole S. (2006). Nonword repetition and word learning: The nature of the relationship. Appl. Psycholinguist..

[33] Pierce L., Genesee F., Delcenserie A., Morgan G. (2017). Variations in phonological working memory: Linking early language experiences and language learning outcomes. Appl. Psycholinguist..

[34] Cheung H. (1996). Nonword span as a unique predictor of second-language vocabulary learning. Dev. Psychol..

[35] French L. (2006). Phonological Working Memory and Second Language Acquisition: A Developmental Study of Francophone Children Learning English in Quebec.

[36] Service E., Simard D., Schwieter J., Wen Z. (2022). How measures of working memory relate to L2 vocabulary. The Cambridge Handbook of Working Memory and Language.

[37] Ellis N.C., Sinclair S. (1996). Working memory in the acquisition of vocabulary and syntax. Q. J. Exp. Psychol..

[38] Foster P., Bolibaugh C., Kotula A. (2014). Knowledge of nativelike selections in an L2: The influence of exposure, memory, age of onset and motivation in foreign language and immersion settings. Stud. Second. Lang. Acquis..

[39] Martin K.I., Ellis N.C. (2012). The roles of phonological STM and working memory in L2 grammar and vocabulary learning. Stud. Second. Lang. Acquis..

[40] Skrzypek A. (2009). Phonological short-term memory and L2 collocational development in adult learners. EUROSLA Yearb..

[41] Hawkins J.A., Schwieter J., Wen Z. (2022). Have grammars been shaped by working memory and if so, how?. The Cambridge Handbook of Working Memory and Language.

[42] Kallens P.C., Christiansen M.H. (2022). Models of language and multiword expressions. Front. Artif. Intell..

[43] O’Brien I., Segalowitz N., Collentine J., Freed B. (2006). Phonological memory and lexical, narrative, and grammatical skills in second language oral production by adult learners. Appl. Psycholinguist..

[44] O’Brien I., Segalowitz N., Collentine J., Freed B. (2007). Phonological memory predicts L2 oral fluency gains in adults. Stud. Second. Lang. Acquis..

[45] French L.M., O’Brien I. (2008). Phonological memory and children’s second language grammar learning. Appl. Psycholinguist..

[46] Juffs A., Harrington M. (2011). Aspects of working memory in L2 learning. Lang. Teach..

[47] Wen Z., Li S., Schwieter J.W., Benati A. (2019). Working memory in L2 learning and processing. The Cambridge Handbook of Language Learning.

[48] Williams J.N., Gass S., Mackey A. (2012). Working memory and SLA. Handbook of Second Language Acquisition.

[49] Baddeley A.D., Schwieter J., Wen Z. (2022). Working memory and challenges of language. The Cambridge Handbook of Working Memory and Language.

[50] Pratto F., John O.P. (1991). Automatic vigilance: The attention-grabbing power of negative social information. J. Personal. Soc. Psychol..

[51] Allen R.J., Baddeley A.D., Thorn A., Page M. (2009). Working memory and sentence recall. Interactions between Short-Term and Long-Term Memory in the Verbal Domain.

[52] Baddeley A.D., Hitch G.J., Bower G.H. (1974). Working memory. The Psychology of Learning and Motivation: Advances in Research and Memory.

[53] Yang T., Gathercole S.E., Allen R.J. (2014). Benefit of enactment over oral repetition of verbal instruction does not require additional working memory during encoding. Psychon. Bull. Rev..

[54] Allen R.J., Waterman A.H., Yang T.X., Jaroslawska A.J., Logie R.H., Wen Z., Gathercole S.E., Cowan N., Engle R.W. (2022). Working memory in action: Remembering and following instructions. Memory in Science for Society: There is Nothing as Practical as a Good Theory.

[55] Conway A.R.A., Moore A.B., Kane M.J. (2009). Recent trends in the cognitive neuroscience of working memory. Cortex.

[56] Engle R.W. (2002). Working memory capacity as executive attention. Curr. Dir. Psychol. Sci..

[57] Engle R.W. (2018). Working memory and executive attention: A revisit. Perspect. Psychol. Sci..

[58] Conway A., Kane M., Bunting M., Hambrick D., Wilhelm O., Engel R. (2005). Working memory span tasks: A methodological review and user’s guide. Psychon. Bull. Rev..

[59] Burgoyne P., Jessie A., Martin D., Mashburn C.A., Tsukahara J.S., Draheim C., Engle R.W., Schwieter J., Wen Z. (2022). Measuring individual differences in working memory capacity and attention control and their contribution to language comprehension. The Cambridge Handbook of Working Memory and Language.

[60] Shin J., Hu Y., Schwieter J., Wen Z. (2022). A methodological synthesis of working memory tasks in L2 research. The Cambridge Handbook of Working Memory and Language.

[61] Wen Z., Juffs A., Winke P., Winke P., Brunfaut T. (2021). Measuring working memory. The Routledge Handbook of Second Language Acquisition and Testing.

[62] Daneman M., Carpenter P.A. (1980). Individual differences in working memory and reading. J. Verbal Learn. Verbal Behav..

[63] Waters G.S., Caplan D. (1996). The measurement of verbal working memory and its relation to reading comprehension. Q. J. Exp. Psychol..

[64] Sweller J., Roussel S., Tricot A., Schwieter J., Wen Z. (2022). Cognitive load theory and instructional design for language learning. The Cambridge Handbook of Working Memory and Language.

[65] Novick J.M., Bunting M.F., Dougherty M.R., Engle R.W. (2019). Cognitive and Working Memory Training: Perspectives from Psychology, Neuroscience, and Human Development.

[66] Sáfár A., Kormos J. (2008). Revisiting problems with foreign language aptitude. Int. Rev. Appl. Linguist. Lang. Teach..

[67] Owen A.M., McMillan K.M., Laird A.R., Bullmore E. (2005). N-back working memory paradigm: A meta-analysis of normative functional neuroimaging studies. Hum. Brain Mapp..

[68] Daneman M., Merikle P.M. (1996). Working memory and language comprehension: A meta-analysis. Psychon. Bull. Rev..

[69] Peng P., Barnes M., Wang C., Wang W., Li S., Swanson H.L., Dardick W., Tao S. (2018). A meta-analysis on the relation between reading and working memory. Psychol. Bull..

[70] Li S., Loewen S., Sato M. (2017). Cognitive differences and ISLA. The Routledge Handbook of Instructed Second Language Acquisition.

[71] Linck J.A., Osthus P., Koeth J.T., Bunting M.F. (2014). Working memory and second language comprehension and production: A meta-analysis. Psychon. Bull. Rev..

[72] Shin J. (2020). A meta-analysis of the relationship between working memory and second language reading comprehension: Does task type matter?. Appl. Psycholinguist..

[73] Miyake A., Friedman N.P., Emerson M.J., Witzki A.H., Howerter A., Wager T. (2000). The unity and diversity of executive functions and their contributions to complex “frontal lobe” tasks: A latent variable analysis. Cogn. Psychol..

[74] Miyake A., Friedman N.P. (2012). The nature and organization of individual differences in executive functions: Four general conclusions. Curr. Dir. Psychol. Sci..

[75] Bunting M.F., Cowan N., Saults J.S. (2006). How does running memory span work?. Q. J. Exp. Psychol..

[76] Calvo N., Ibáñez A., García A.M. (2016). The Impact of Bilingualism on Working memory: A Null Effect on the Whole May Not Be So on the Parts. Front. Psychol..

[77] Gass S., Lee J., Schmid M., Lowie W. (2011). Working memory capacity, inhibitory control, and proficiency in a second language. From Structure to Chaos: Twenty Years of Modeling Bilingualism: In Honor of Kees De Bot.

[78] Skehan P., Schwieter J., Wen Z. (2022). Working memory and L2 speaking tasks. The Cambridge Handbook of Working Memory and Language.

[79] Burgoyne A.P., Engle R.W. (2020). Attention control: A cornerstone of higher-order cognition. Curr. Dir. Psychol. Sci..

[80] Draheim C., Pak R., Draheim A.A., Engle R.W. (2022). The role of attention control in complex real-world tasks. Psychon. Bull. Rev..

[81] Green D.W., Abutalebi J. (2013). Language control in bilinguals: The adaptive control hypothesis. J. Cogn. Psychol..

[82] Dong Y., Li P. (2020). Attentional control in interpreting: A model of language control and processing control. Biling. Lang. Cogn..

[83] In’nami Y., Hijikata Y., Koizumi R. (2021). Working memory capacity and L2 reading: A meta-analysis. Stud. Second. Lang. Acquis..

[84] Wen Z., Jackson D.D., Li S., Hiver P., Papi M. (2022). Working memory. Routledge Handbook of Second Language Acquisition and Individual Differences.

[85] Wen Z., Wen Z., Skehan P., Biedroń A., Li S., Sparks R. (2019). Working memory as language aptitude: The Phonological/Executive Model. Language Aptitude: Advancing Theory, Testing, Research and Practice.

[86] Wen Z., Skehan P. (2021). Stages of acquisition and the P/E Model of working memory: Complementary or contrasting approaches to foreign language aptitude?. Annu. Rev. Appl. Linguist..

[87] Purcell J., Rapp B., Martin R.C. (2021). Distinct neural substrates support phonological and orthographic working memory: Implications for theories of working memory. Front. Neurol..

[88] Christiansen M.H., Chater N. (2017). Towards an integrated science of language. Nat. Hum. Behav..

[89] Juffs A. (2017). The importance of grain size in phonology and the possibility that phonological working memory is epiphenomenal. Appl. Psycholinguist..

[90] Schwering S.C., MacDonald M.C. (2020). Verbal working memory as emergent from language comprehension and production. Front. Hum. Neurosci..

[91] Lu B., Wen Z., Schwieter J., Zhisheng W. (2022). Short-term and working memory capacity and the language device: Chunking and parsing complexity. The Cambridge Handbook of Working Memory and Language.

[92] Chomsky N. (1965). Aspects of the Theory of Syntax.

[93] Hawkins J.A. (2004). Efficiency and Complexity in Grammars.

[94] O’Grady W. (2017). Working memory and language: From phonology to grammar. Appl. Psycholinguist..

[95] Liu H. (2008). Dependency distance as a metric of language comprehension difficulty. J. Cogn. Sci..

[96] Gibson E., Futrell R., Piantadosi S.T., Dautriche I., Mahowald K., Bergen L., Levy R.P. (2019). How efficiency shapes human language. Trends Cogn. Sci..

[97] MacDonald M.C. (2016). Memory limitations and chunking are variable and cannot explain language structure. Behav. Brain Sci..

[98] Postle B.R. (2006). Working memory as an emergent property of the mind and brain. Neuroscience.

[99] Rączy K., Orzechowski J. (2021). When working memory is in a mood: Combined effects of induced affect and processing of emotional words. Curr. Psychol..

[100] Gregersen T., Mercer S. (2022). Routledge Handbook for the Psychology of Language Learning.

[101] Arnold M.B. (1960). Emotion and Personality.

[102] Mikels J.A., Reuter-Lorenz P.A., Pashler H. (2013). Emotion and working memory. Encyclopedia of the Mind.

[103] Zhao M., Jia W., Yang D., Nguyen P., Nguyen T.A., Zeng Y. (2020). A tEEG framework for studying designer’s cognitive and affective states. Des. Sci..

[104] Estes Z., Adelman J.S. (2008). Automatic vigilance for negative words in lexical decision and naming: Comment on Larsen, Mercer, and Balota. Emotion.

[105] Ohman A., Mineka S. (2001). Fears, phobias, and preparedness: Toward an evolved module of fear and fear learning. Psychol. Rev..

[106] Garrison K.E., Schmeichel B.J. (2019). Effects of emotional content on working memory capacity. Cogn. Emot..

[107] Storbeck J., Maswood M. (2016). Happiness increases verbal and spatial working memory capacity, sadness does not: Emotion, working memory, and executive control. Cogn. Emot..

[108] Yang H., Sujin Y., Alice M.I. (2013). Positive affect improves working memory: Implications for controlled cognitive processing. Cogn. Emot..

[109] Storbeck J., Davidson N.A., Dahl C.F., Blass S., Yung E. (2015). Emotion, working memory task demands and individual differences predict behavior, cognitive effort and negative affect. Cogn. Emot..

[110] Yang J., Yang L., Quan H., Zeng Y. (2021). Implementation Barriers: A TASKS Framework. J. Integr. Des. Process Sci..

[111] Nguyen T.A., Zeng Y. (2014). A physiological study of relationship between designer’s mental effort and mental stress during conceptual design. Comput. Aided Des..

[112] Nguyen T.A., Zeng Y. (2012). A theoretical model of design creativity: Nonlinear design dynamics and mental stress-creativity relation. J. Integr. Des. Process Sci...

[113] Nguyen T.A., Zeng Y. (2017). Effects of stress and effort on self-rated reports in experimental study of design activities. J. Intell. Manuf..

[114] Barker R.M., Bialystok E. (2019). Processing differences between monolingual and bilingual young adults on an emotion *N-back* task. Brain Cogn..

[115] Antoniou M. (2019). The advantages of bilingualism debate. Annu. Rev. Linguist..

[116] Luk G., Bialystok E. (2013). Bilingualism is not a categorical variable: Interaction between language proficiency and usage. J. Cogn. Psychol..

[117] Novitskiy N., Shtyrov Y., Myachykov A. (2019). Conflict resolution ability in late bilinguals improves with increased second-language proficiency: ANT evidence. Front. Psychol..

[118] Aben B., Stapert S., Blokland A. (2012). About the distinction between working memory and short-term memory. Front. Psychol..

[119] D’Esposito M., Postle B.R., Ballard D., Lease J. (1999). Maintenance versus manipulation of information held in working memory: An event-related fMRI study. Brain Cogn..

[120] Luo Y., Qin S., Fernandez G., Zhang Y., Klumpers F., Li H. (2014). Emotion perception and executive control interact in the salience network during emotionally charged working memory processing. Hum. Brain Mapp..

[121] Zhang P.C., Leng Y., Lu J.M. (2017). The empirical research on the influence of emotional experience and cognitive load on the working memory. Psychol. Explor..

[122] Grimm S., Weigand A., Kazzer P., Jacobs A.M., Bajbouj M. (2012). Neural mechanisms underlying the integration of emotion and working memory. Neuroimage.

[123] Schweizer S., Grahn J., Hampshire A., Mobbs D., Dalgleish T. (2013). Training the emotional brain: Improving affective control through emotional working memory training. J. Neurosci..

[124] Engen H.G., Anderson M.C. (2018). Memory control: A fundamental mechanism of emotion regulation. Trends Cogn. Sci..

[125] Ladouceur C.D., Silk J.S., Dahl R.E., Ostapenko L., Kronhaus D.M., Phillips M.L. (2009). Fearful faces influence attentional control processes in anxious youth and adults. Emotion.

[126] Plutchik R. (2001). The nature of emotions: Human emotions have deep evolutionary roots, a fact that may explain their complexity and provide tools for clinical practice. Am. Sci..

[127] (2002). Psychology Software Tools. E-Prime.

[128] Conway A.R.A., Engle R.W. (1996). Individual differences in working memory capacity: More evidence for a general capacity theory. Memory.

[129] Engle R.W., Kane M.J., Tuholski S.W., Miyake A., Shah P. (1999). Individual differences in working memory capacity and what they tell us about controlled attention, general fluid intelligence, and functions of the prefrontal cortex. Models of Working Memory.

[130] Ashcraft M.H., Kirk E.P. (2001). The relationships among working memory, math anxiety, and performance. J. Exp. Psychol. Gen..

[131] Friedman N.P., Miyake A. (2004). The reading span test and its predictive power for reading comprehension ability. J. Mem. Lang..

[132] Miyake A. (2001). Individual differences in working memory: Introduction to the Special Section. J. Exp. Psychol. Gen..

[133] Pieroni L., Rossi-Arnaud C., Baddeley A.D., Vandierendonck A., Szmalec A. (2011). What can symmetry tell us about working memory?. Spatial Working Memory.

